# Distinct Lipid Compositions in Seawater and the Sea Surface Microlayer Measured with Liquid Chromatography and High-Resolution Mass Spectrometry

**DOI:** 10.1021/acsearthspacechem.6c00084

**Published:** 2026-07-30

**Authors:** Amber E. Birt, Daniel Ammer, Felix E. Agblemanyo, Andrew S. Wozniak, Amanda A. Frossard

**Affiliations:** † Department of Chemistry, University of Georgia, Athens, Georgia 30606, United States; ‡ School of Marine Science and Policy, 522742 University of Delaware, Lewes, Delaware 19958, United States

**Keywords:** sea surface microlayer, organic enrichment, lipids, liquid chromatography mass spectrometry, phospholipids, glycolipids

## Abstract

The sea surface microlayer (SML) plays an important role in the air–sea exchange of gases and particles. Surfactants accumulate at and modulate the properties of the SML, but specific chemical and physical properties driving variability in their distribution in the SML remain unclear. Lipids are a type of surfactant in the ocean and are biomarkers for dissolved organic matter (DOM) sources. We collected paired SML and associated subsurface seawater (SSW) samples over three time points in the North Atlantic Ocean in September 2022 and used liquid chromatography and high-resolution mass spectrometry (LC–MS) to identify 163 and 189 lipids in the SML and SSW, respectively. Of those, just 33 were identified in both layers. Lipids were classified as glycolipids, phospholipids, triglycerols, diacylglycerides, monoacylglycerides, and betaine lipids, as well as lipid byproducts, including free fatty acids and polyunsaturated aldehydes. Enrichment factors for each lipid class show overall enrichment in the SML (average of 1.68 ± 1.43 for all classes (*n* = 28) over all times (*n* = 3)), varying throughout the day (3.33 ± 4.13 for Night, 0.79 ± 1.76 for Afternoon, and 0.92 ± 1.61 for Evening). Based on these enrichments and ratios of specific lipids, we show that the SML and SSW are unique microenvironments, with bacterial lipids and degradation products enriched in the SML relative to the SSW. Bacteria and DOM degradation processes may be important for shaping the lipid and thus surfactant composition in the SML. Determining the types of lipids and their sources and degradation processes in the SML may improve our understanding of the sources and properties of surfactants and thus their influence on air–sea exchange processes.

## Graphical abstract

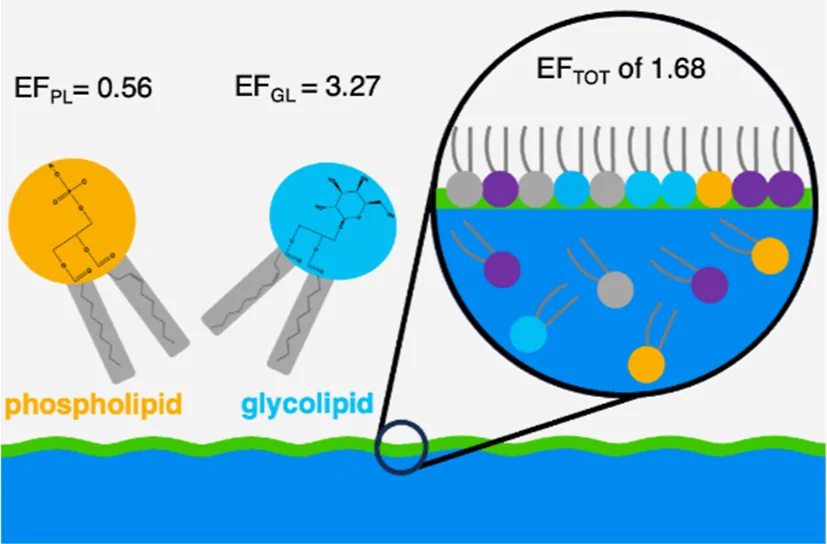

## 1 Introduction

The ocean covers more than 70% of the Earth’s surface, and gases, such as CO_2_, and particles can be exchanged between the ocean and the atmosphere.^1–3^ Air–sea exchange occurs at the surface microlayer (SML), which is 10s to 100s of μm thick and is enriched with dissolved organic matter (DOM) and surface-active organics.^2^ The properties of surface-active organics in the SML can modulate production and chemical and physical properties of sea spray aerosol particles^4^ as well as air–sea gas exchange.^5–7^ Uncertainties in our understanding of the physical and chemical properties of surface-active organics in the SML, including how oceanographic conditions and processes govern their variability, can lead to biases in estimates of air–sea exchanges of gases and particles.^5,7^

Surfactants accumulate at the air–water interface^8–11^ due to their amphiphilic structure, with both hydrophobic and hydrophilic functional groups.^12^ Surfactants can be transported to the SML from the underlying seawater by rising bubbles.^13^ Enrichment of surfactants in the SML can be variable, depending on the biological activity in the seawater or the progression of a phytoplankton bloom.^14^ Bubble bursting emits a fraction of the surfactant compounds from the seawater into the atmosphere as aerosols and the rest remain at the air–sea interface, in the SML.^13^ Some lipids are biologically produced DOM abundant in the SML and may thus be considered candidate surfactants useful for understanding air–sea exchange processes.^15^ Despite their low concentrations in seawater compared to carbohydrates and proteins,^16^ lipids, with their characteristic amphiphilic structures, are considered surfactants and play an important role in forming and stabilizing the SML due to their high surface activity.^17,18^ Lipids have also been observed to have higher enrichment in aerosol particles emitted from the ocean than amino acids and carbohydrates.^19^

Lipids make up cell membranes, including those of bacteria, and contribute to key biological processes in the marine environments.^20^ Intact polar lipids are a diverse suite of molecules made up of a glycerol backbone and 1 to 3 fatty acid chains connected by an ether or an ester bond, making them surface-active (Table 1).^16,21,22^ Lipids are grouped into glycolipids, which contain carbohydrates, and phospholipids, which contain phosphates and various head groups (e.g., a choline) (Table 1).^22^ Lipids can have other defining features, such as sulfur functional groups (sulfolipids) or only one fatty acid chain (lysophospholipids), as listed in Table 1. The interfacial properties of lipids, which may control their enrichment in the SML,^18^ vary among lipid classes, depending on specific features, such as carbon number, chain length, branching, and headgroup identity.^16,22^ For example, increasing carbon chain length can increase surface activity.^23,24^ Head groups can affect the surface activity of the lipids as well. Certain nonpolar lipids, such as triglycerol (TG; grouped as “Other” in Table 1), result in higher surface activity compared to phospholipids (Table 1), such as phosphatidylethanolamine (PE) and phosphatidylglycerol (PG).^16^ Monogalactosyldiacylglycerol (MGDG) is one of the most common glycolipids (Table 1) in plankton,^22,25^ and it has a sugar-containing headgroup, which increases its solubility and limits surface-activity. Betaine lipids do not contain phosphorus, are zwitterionic, and contain a betaine polar headgroup.^22,26^ Previous work has identified the betaine lipid diacylglyceryltrimethylhomoserine (DGTS) in seawater.^27^ Archaeal lipids have fatty-acid chains that are ether-linked (e.g., PE O) instead of ester-linked (e.g., PE), which is a characteristic that differs from most bacterial and eukaryal lipids.^28^

**Table 1:** Lipid Classes Identified in This Study with Associated Acronyms, Linkage, Type, Average Number of Carbons, and Number of Total Identifications, Separated by General Lipid Group as Glycolipids, Phospholipids, or Other Lipids

acronym	lipid class name	linkage	type	average number of carbons	number identified
Glycolipids					
DGDG	digalactosyldiacylglycerol	ester		27.9 ± 9.0	10
MGDG	monogalactosyldiacylglycerol	ester		25.2 ± 11.1	32
SQDG	sulfoquinovosyldiacylglycerol	ester	sulfolipid	22.5 ± 14.8	15
SQMG	sulfoquinovosylmonoacylglycerol	ester	sulfolipid	21.9 ± 10.8	31
Phospholipids					
CL	cardiolipin	ester		22.7 ± 8.5	6
LPA O	monoalkylglycerophophate	ether	lysophospholipid	18 ± 11.5	5
LPC	lysophosphatidylcholine	ester	lysophospholipid	25.3 ± 10.2	4
LPC O	monoalkylglycerophosphocholine	ether	lysophospholipid	27 ± 8.3	14
LPE O	monoalkylglcerophosphoethanolamine	ether	lysophospholipid	31.9 ± 7.6	11
LPG O	monoalkylglycerophosphoglycerol	ether	lysophospholipid	18 ± 7.2	11
LPI	monoacylglycerophosphoinositol	ester	lysophospholipid	14 ± 0	3
LPI O	monoalkylglycerophosphoinositol	ether	lysophospholipid	13.2 ± 6.8	18
PA O	alklacylglycerophosphate	ether		14 ± 3.9	7
PC	phosphatidylcholine	ester		28.5 ± 12.0	17
PC O	dialkylglycerophosocholine	ether		20.4 ± 11.3	16
PE	phosphatidylethanolamine	ester		27.8 ± 14.3	5
PE O	dialkylglycerophosphoethanolamine	ether		19 ± 9.8	6
PG	phosphatidylglycerol	ester		17.4 ± 6.2	5
PG O	dialkylglycerolphosphoglycerol	ether		24 ± 6.6	7
PI	phosphatidylinositol	ester		21.1 ± 8.6	19
PI O	dialkylglycerolphosphoinositol	ether		17.9 ± 5.0	8
PS	phosphatidylserine	ester		24.3 ± 10.2	4
Other Lipids					
DG	diacylglyceride	ester		19.2 ± 8.8	9
DGTS	diacylglyceryltrimethylhomoserine	ether	betaine lipid	25.4 ± 9.2	16
MG	monoacylglyceride	ester		21.1 ± 9.9	12
MG O	monoalkylglycerol	ether		22 ± 18.1	6
TG	triacylglycerol	ester		25.9 ± 10.9	48
TG O	alkyldiacylglyceride	ether		17.2 ± 8.1	45

Studying lipids and their classes can be useful when analyzing the carbon cycle of the ocean,^29^ and both phytoplankton and microbial communities participate in the carbon cycle of the surface ocean.^14^ For example, phytoplankton contribute ∼50% of the Earth’s carbon fixation, and ∼15–49% of the carbon in planktonic cells come from lipids, including those that make up their cell membranes.^30–32^ Importantly, the composition of the lipid pool in the ocean changes depending on the conditions and biological community present, allowing for their use as biomarkers.^33^ Sources of DOM in the SML can be estimated by understanding the biological processes and sources of lipids and their classes.^34,35^ Common sources for lipids in DOM include cell lysis, direct release from phytoplankton, and metabolic processes.^33^ Polar lipids are labile when outside living cells in the ocean and can lose their headgroups relatively quickly (∼ hours or ∼ days, depending on environmental factors).^36^ Therefore, they are unlikely to exist outside living cells, thus making them good biomarkers for living organisms (or DOM freshly released from living organisms).^36–40^ The intact polar lipid PE is an indicator of bacterial lipids since it is predominately found in the membranes of bacteria,^41^ and PG is an important phospholipid found in phytoplankton membranes and an indicator of potential microalgal sources.^42^

Marine lipids can be produced by abiotic and biotic processes.^43^ Exposure to solar radiation and the resulting photochemical production and degradation of specific molecules are important abiotic processes in the ocean. Mono- and diacylglycerides (MG and DG) are indicative of degradation and considered to be lipid breakdown products.^35,44^ The degradation level of marine lipids can be calculated using the Lipolysis Index.^44^ Higher degradation is associated with a higher Lipolysis Index, whereas the presence of lipids that are resistant to degradation have lower values.^16^ Lysophospholipids are degradation products of phospholipids with a single fatty acid chain.^45^ Other possible degradation products of lipids and polyunsaturated fatty acids include fatty acid chains that are free floating in the ocean (e.g., free fatty acids)^35,44^ and polyunsaturated aldehydes, which are bioactive molecules released by phytoplankton.^46^ Metabolic reserves can be inferred by the presence of TG, storage molecules in phytoplankton.^34,47^ A diel trend of TG accumulation as a result of photochemistry in laboratory cultures of phytoplankton was observed previously.^48–50^ This accumulation can also be due to phytoplankton growth under unfavorable conditions, such as nutrient deprivation.^51^ The depletion of nutrients such as phosphorus and inorganic nitrogen were measured in the surface ocean,^52^ which can affect lipid composition in phytoplankton and cyanobacteria.^22^ Nutrient depletion can cause organisms to adapt and produce non-phosphorus-containing molecules such as betaine lipids or glycolipids (e.g., sulfoquinovosyldiacylglycerol (SQDG)) instead of phospholipids (e.g., phosphatidylcholine (PC) and PG).^34,53–57^ Cyanobacteria can substitute PG for SQDG,^56^ and betaine lipids are typically substituted for PC in phytoplankton under phosphorus limiting conditions.^58,59^

Given the diversity of molecular structures and associated interfacial properties as well as their source- and process-specific properties, the study of intact polar lipids has potential for providing further insights into SML properties. Previous studies observed fatty acids in DOM and surfactants in sea foams,^60^ the SML, and subsurface seawater (SSW)^61^ using mass spectrometry (MS).^15,62^ Additionally, recent mesocosm studies show varying SML enrichment of DOM that changes with phytoplankton activity during a bloom.^14^ While Iatroscan thin layer chromatography has been used to determine lipid composition in the SML,^16^ it is only with recent advances in MS that the diverse suite of intact polar lipids has been characterized in seawater DOM.^21,27^ The signals of some lipid classes, when present at low concentrations, can be suppressed by those of more concentrated lipid classes. Therefore, fractionation techniques, such as liquid chromatography (LC), are used to analyze complex samples and increase the number of lipids identified.^63^ Studies used LC to separate surface-active marine compounds,^62,64–71^ including fatty acids, in generated marine aerosols^15^ and DOM in the SML and SSW.^62^ One study measured intact polar lipids in the eastern subtropical South Pacific.^27^ With structural elucidation, individual intact polar lipids can be identified^72,73^ allowing for the exploitation of the source- and process-specific information they hold.

To better understand the processes that govern surfactant abundances at the SML, we (i) identified lipids, free fatty acids, and polyunsaturated aldehydes in the SML and SSW collected in the North Atlantic Ocean at multiple times of day and after irradiation; (ii) calculated enrichment factors of lipid classes and measured the surface tension of organics extracted from the SML and SSW; (iii) determined the contribution of lipids to the interfacial properties of the SML; and (iv) inferred potential sources of lipids, surfactants, and DOM in the SML and SSW.

## 2 Methods

### 2.1 SML and SSW Collection

The SML and SSW were collected throughout the day on September 1, 2022, onboard the R/V Hugh R. Sharp during the Surfactants at the Ocean–Atmosphere Physical Interface cruise in the North Atlantic Ocean. Additional details about the project are included in Agblemanyo et al.^74^. Samples were collected from a hydrographic region just past the edge of the ocean shelf, west of the Gulf Stream, referred to as the Continental Slope site (located at 37.83°N, 73.72°W). This region is ∼130 km from land with minimal influence from coastal waters or terrestrial runoff.

The SML and SSW were collected at three times throughout the day: Night (02:39–05:04 ET), Afternoon (13:00–13:55 ET), and Evening (18:34–20:06 ET). The Night sample volumes were twice as large as the others so that half could be allocated to the irradiation experiments (Irradiated). Hereafter, we discuss the Night, Afternoon, Evening, and Irradiated samples from the SML and SSW (Table S1).

The SML was collected using a modified version of the glass plate method^61,75^ as described by Agblemanyo et al.^74^ Briefly, three glass plates were attached to a rosette frame, which was lowered vertically into the water off the stern of the ship using the A-frame. Once the sampler was secure on the ship, the SML was squeegeed off the glass plates, using acid-washed (1 M HCl), Teflon blades into precleaned (1 M HCl) polycarbonate sample bottles. This procedure was continued until a ∼1.5 L of SML water was collected or after ∼2 h of sampling. The average drip time and number of dips for each sample were recorded (Table S1).^74^ The Afternoon and Evening SML samples discussed herein required 30 and 38 dips, respectively. 63 dips were required for the Night SML sample to collect extra volume for the irradiation experiment. The average SML sample thickness across these three samples was 13.38 ± 2.06 μm.^74^ Blank samples replicating the sampling procedure were collected after the glass plates were cleaned with ultrapure water (18.2 MΩ·cm) and 1 M HCl. Ultrapure water was then poured onto the plates and squeegeed into the collection funnel and bottle until a volume of ∼1.5 L was reached.^74^

The paired SSW samples were collected directly following each SML sampling by pumping water with precleaned (1 M HCl) Teflon tubing from ∼0.5 m depth into precleaned (1 M HCl) polycarbonate bottles until ∼1.5 L of water was collected.^74^ SSW blank samples replicating sample processing procedures were collected with ultrapure water and handled the same as the samples.

Immediately after sampling, all SML, SSW, and blank samples were filtered with precombusted (450 °C, 4 h) 0.7 μm glass fiber filters (GF/F, 47 mm) to remove the particulates and retain the DOM.^76^ Samples were then distributed into precleaned bottles for extraction or irradiation, as described later. For all sampling times, two 300 mL and two 100 mL aliquots of SML and SSW were transferred to precleaned amber glass bottles for further processing and analyses. For the Night sampling, an additional two 300 mL and two 100 mL aliquots of SML and SSW were frozen and transported to the laboratory for irradiation. Measured chlorophyll-a concentration, sea surface temperature, and wind speed throughout the project are reported by Agblemanyo et al.,^74^ and values for these samples are included in Table S1.

### 2.2 Irradiation of Seawater

The extra Night samples that were saved for irradiation experiments were thawed at room temperature overnight in the laboratory and then transferred to temperature-controlled jacketed beakers, with recirculating cooling water (Figure S1). Irradiation experiments were also performed using blanks of ultrapure water that were subjected to the same preprocessing as the SML and SSW samples.

Two beakers containing 400 mL of SML, SSW, or blanks were covered with quartz glass lids to prevent evaporation and irradiated for 4 h in a solar simulator, while being stirred continuously (Figure S1).^77^ The solar simulator (Suntest) was equipped with a 1.5 kW xenon lamp fitted with an infrared-filtering glass window to simulate sunlight at Earth’s surface.^77^ The radiative flux of the solar simulator was calculated to be equivalent to ∼10.5 h of noontime sun. The Irradiated water was then processed using the same methods as the original Afternoon, Evening, and Night SML and SSW samples.

### 2.3 Solid Phase Extraction of Organic Molecules

Two different solid phase extraction (SPE) cartridges, ENVI-18 (C18 sorbent material, 0.5 g bed weight, MilliporeSigma) and ENVI-Carb (graphitized carbon sorbent material, 0.5 g bed weight, MilliporeSigma) cartridges were used to collect large, organic molecules from the filtered water.^78^ Using high-resolution MS analyses, ENVI-18 cartridges have high extraction efficiencies for anionic (85%) and nonionic (60%) surfactants, and ENVI-Carb cartridges have high extraction efficiencies for cationic surfactants (75%).^78^ While extraction efficiencies for specific lipids may differ, compared to those of the surfactant standards reported by Burdette and Frossard,^78^ lipid structures are generally similar to each other, and the lipids identified in this study are considered surfactants. Because the same methods are used for all samples, any lipid-specific extraction efficiencies are applied systematically, and comparisons between samples are consistent. Additionally, C18 SPE cartridges were used to extract DOM in other studies.^79^

For MS analyses, the ENVI-18 and ENVI-Carb SPE cartridges were conditioned with 6 mL of acetonitrile (MilliporeSigma) and rinsed with 12 mL of ultrapure water.^78^ 300 mL of the sample was passed through each cartridge with a targeted flow rate between 1 and 2 mL min^–1^. After processing the sample, 4 mL of 0.1% triethylamine (MilliporeSigma) in ultrapure water was used to rinse the sample bottle and processed through the cartridge.^78^ The cartridges were then washed with 8 mL of 0.1% triethylamine and dried under low vacuum.^78^ The cartridges were wrapped in aluminum foil and frozen until their elution and further processing at the University of Georgia (UGA) (Section 2.5).

For surface tension measurements, samples were processed similarly to those for MS analyses but using only the ENVI-18 cartridges, 100 mL samples, and ultrapure water instead of 0.1% triethylamine to rinse the bottles and wash the cartridges. The dried cartridges were wrapped in aluminum foil and frozen until their elution and processing at UGA (Section 2.4).

### 2.4 Surface Tension Measurements

From the ENVI-18 cartridges for surface tension analyses, organic compounds were eluted using 8 mL of acetonitrile and collected into precleaned vials.^78^ Dry nitrogen gas (Airgas; >99% purity) was used to evaporate the acetonitrile, leaving only the organic extracts. These extracts were rehydrated with 40 μL of ultrapure water and analyzed using a pendant drop tensiometer.^80^ A syringe with a microneedle tip of either 0.30 mm or 0.51 mm was used to suspend the droplet for samples with surface tensions less than or greater than 44 mN m^–1^, respectively. Each droplet was allowed to stabilize and equilibrate for 30 s^81,82^ Surface tension measurements were then made over 10 s intervals at a rate of 5 frames per second^83,84^ and averaged for each droplet. Measurements of 3–5 droplets were averaged for each sample. The surface tension of the 40 μL rehydrated samples is referred to as the surface tension minimum. In standard surface tension isotherms, the surface tension minimum is the first point on the isotherm, before dilution.

### 2.5 Characterization of Organic Extracts and Identification of Lipids

Organics were eluted from the ENVI-18 and ENVI-Carb cartridges, using 4 mL of acetonitrile for each and collected separately into precleaned vials.^78^ Dry nitrogen gas was used to evaporate the acetonitrile, leaving the dried organic compounds.^78^ A 1:1 mixture of acetonitrile and ultrapure water was used to rehydrate the extracts to a total volume of 300 μL.

LC–MS analysis was performed on the rehydrated extracts at the Proteomics and Mass Spectrometry (PAMS) facility at UGA. 23.8 ng of Genistein (MilliporeSigma) and 84.6 ng of Reserpine (SCIEX) were added to the rehydrated samples for ENVI-18 and ENVI-Carb, respectively, as internal standards with 5 μM in each.^78^ A volume of 2–5 μL of each sample was injected into an Elute ultrahigh performance LC system (Bruker). A Luna C8(2) LC column (100 × 2.0 mm) with a particle size of 3 μm and a pore size of 100 Å (Phenomenex) was used with a separate gradient method for each ionization mode. A flow rate of 0.2 mL min^–1^ and a mobile phase of ultrapure water and acetonitrile were used with the gradient profiles (Table S2). The chromatograms were split into Most Polar, Intermediate Polarity, and Least Polar based on retention times,^62^ as described in Table S2.

ENVI-Carb rehydrated extracts were run in positive ionization mode, and ENVI-18 rehydrated extracts were run in negative ionization mode.^78^ The LC system was coupled with an electrospray ionization quadrupole time-of-flight mass spectrometer (ESI-Q-TOF-MS; Impact II, Bruker). Data were collected by using a capillary voltage of 4.5 kV for positive ionization and 3.5 kV for negative ionization. Both intact (MS1) and fragmented (MS2) spectra were collected.

The instrument software (DataAnalysis, Bruker) was used to process the high-resolution line spectra that were collected. A calibration solution of common organic standards was added before each injection to confirm that a mass accuracy within 1 ppm was measured. The data were then converted to an open-source format (.mzML) using MSConvert (ProteoWizard),^85^ and the files were uploaded into MZmine3 ^86^ for feature extraction and lipid assignment.

Mass detection was performed, and a feature table was built using the Automated Data Analysis Pipeline Chromatogram builder module.^87,88^ For MS1 chromatogram building, a minimum intensity of 1000 and a scan-to-scan tolerance of 5 ppm were used as a peak detection threshold. MS1 chromatogram building creates an extracted ion chromatogram for masses in MS1 spectra.^88^ Additionally, a local minimum resolver was applied to separate overlapping and partially coeluting peaks. A maximum of 5 ppm and a minimum absolute height of 1000 were allowed between the MS1 precursor ion scan and mass-to-charge of the MS2 precursor scan.^86^ Lipids were tentatively identified from the built-in database. Previous studies have found a maximum of 40 carbons and 12 double bonds in the combined fatty acids of intact polar diacylglycerolipids.^27^ However, in order to study TG, which has three fatty acid chains,^89^ lipids were limited to 1–54 carbon atoms and 0–12 double bonds in the combined fatty acid chains, and a tolerance of 5 ppm was used.

Finally, peak intensities and corresponding lipid identifications were exported into a feature table. Any identifications made in the corresponding SML or SSW blanks were removed from the sample identifications, with one blank of each type used for all of the corresponding samples, to be consistent. Surface tensions of the SML, SSW, and Irradiated blanks prior to extraction were all the same as those of ultrapure water. Double identifications were treated as follows: if an identification was made more than once in the same sample and had the same adduct, then the higher intensity peak was kept. However, if the adducts were different, then the intensities were added together.^90^ The positive- and negative-ionization-mode identifications were then combined. Any duplicates in identifications, from identifications made in both ionization modes, were removed from the combined data set.

To identify free fatty acids and polyunsaturated aldehydes in the SML and SSW, the LC–MS data were analyzed following a modified method from Collins et al.^91^ The centroid mass spectral data were preprocessed using xcms^92–94^ and CAMERA,^95^ following Patti et al.^96^ The xcms package was used to process and visualize mass spectral data that had been separated using LC.^92–94^ The method was modified by changing the retention time correction method from ordered bijective interpolated warping (“obiwarp”)^97^ to locally estimated scatterplot smoothing (“loess”)^98^ to align chromatographic peaks across multiple samples and remove outliers.^99^ The features were grouped together across samples before and after retention time correction was performed, based on information in Collins et al.^91^ CAMERA was used to annotate the resulting peaklists using a set of rules such as those involving isotopes and adduct formation.^95^ LOBSTAHS^91^ was then used to identify polyunsaturated aldehydes and free fatty acids. Compound assignments were matched to the LOBSTAHS database with a tolerance of 5 ppm. Only “high confidence” identifications or those without structural isomers or isobars were used.^91^ The peak areas for all free fatty acid and polyunsaturated aldehyde identifications in the samples were then normalized as described in Collins et al.^91^ Any identifications made in the blanks were removed from the associated SML and SSW identifications. Double identifications were treated as follows: if an identification was made more than once in the same sample, the identification with the lower error (in ppm) was kept. The positive and negative ionization mode identifications were combined as described for the lipid identifications.

### 2.6 Enrichment Factors and Lipid Ratios

Enrichment factors of lipids in the SML compared to the SSW (EF_Lipids_) were calculated by dividing the sum of the intensities of identified lipids in the SML by the sum of the intensities of identified lipids in the SSW (eq 1) for each individual lipid class:

$${EF}_{\;Lipids}=\frac{\sum I_{SML}}{\sum I_{SSW}}$$ 1

Semiquantitative estimates of tentative formula identifications are frequently made using signal intensity.^15^ The EF of polyunsaturated aldehydes (EF_PUA_) and the EF of free fatty acids (EF_FFA_) were calculated by dividing the sum of the normalized peak areas of polyunsaturated aldehydes or free fatty acids identified in the SML by the corresponding sum of those identified in the SSW (eq 2):

$${EF}_{\;PUA\;\mathrm{or}\;FFA}=\frac{\sum A_{SML}}{\sum A_{SSW}}$$ 2

Because LOBSTAHS^91^ was used to identify polyunsaturated aldehydes and free fatty acids, resulting peak areas were used here instead of intensities. A value of EF > 1 demonstrates an enrichment in the SML compared to the SSW, while a value of EF < 1 indicates a depletion. Overall EFs are reported as the average of the EFs of the individual lipid classes for each sample.

The Lipolysis Index was calculated as the sum of the intensities of two lipid metabolites (MG and DG) divided by the sum of the intensities of their lipid precursors (TG, MGDG, digalactosyldiacylglycerol (DGDG), SQDG, PG, PE, PC, phosphatidylinositol (PI), phosphatidylserine (PS), and cardiolipin (CL))^57^ (eq 3):^44^

3 LipolysisIndex=(MG+DG)/(TG+MGDG+DGDG+SQDG+PG+PE+PC+PI+PS+CL)

To determine the dominant biomass (phytoplankton or bacterial) in the SML and SSW, PE/PG ratios were calculated by dividing the intensity of PE lipids by that of PG lipids.^42^ To investigate lipid abundances indicative of potential nutrient limitation, the ratio of betaine lipids to PC and the ratio of SQDG to PG were calculated. In this study, the only lipid class identified as a betaine lipid was DGTS; therefore, we report the DGTS/PC ratio. The DGTS/PC and SQDG/PG ratios were calculated by dividing the intensity of DGTS or SQDG by the intensity of PC or PG, respectively.^56^ DGTS/PC and SQDG/PG ratios >1 can indicate nutrient limiting conditions.^56^ The octanol–water partition coefficient (*K*_OW_) was determined for each lipid class.^100^ Identified lipid classes were grouped as hydrophilic (*K*_OW_ < 1), less hydrophobic (*K*_OW_ of 1–3), and more hydrophobic (*K*_OW_ > 3) (Table S3).

## 3 Results and Discussion

### 3.1 Surface-Active Organics Observed in the SML and SSW

Seawater chlorophyll-a concentrations, atmospheric temperatures, and wind speeds during sampling did not vary significantly across the three time points and showed very little to no differences between the conditions during SML sampling and SSW sampling, as shown in Table S1. This confirms that the SML and SSW were paired at each time point and can be directly compared. The wind speeds during sampling were all less than 7 m s^–1^ (Table S1). 8 m s^–1^ is generally considered the wind speed required for breaking waves and generation of sea spray aerosol, indicating that a SML could be present and undisturbed during the sample times. Studies have also shown that the SML is present at wind speeds up to 13 m s^–1^ and will regenerate quickly when disturbed.^101^

Lipids were identified in all eight samples, with a total of 352 lipid identifications, 163 in the SML and 189 in the SSW samples. Previous work shows a similar total number of lipid identifications (373) in open-ocean samples collected at 5 m depth.^102^ Lipids have been observed to be surfactants,^17^ which decrease the surface tension of pure water.^4,103^ The minimum surface tensions for the organic extracts measured herein ranged from 32.3 to 58.2 mN m^–1^ (mean of 39.5 ± 12.5 mN m^–1^) for the SML and 34.8–56.6 mN m^–1^ (mean of 44.8 ± 9.6 mN m^–1^) for the SSW. These minimum surface tensions were all significantly lower than the measured surface tension of pure water (71.4 ± 0.6 mN m^–1^) and the average surface tensions of the SML (69.80 ± 0.09 mN m^–1^) and SSW (69.76 ± 0.6 mN m^–1^) samples prior to extraction. The surface tension minimums and surface tensions prior to extraction are all also lower than the surface tensions of the blanks (70.8 ± 0.4 mN m^–1^ SML blank, 71.1 ± 0.3 mN m^–1^ SSW blank, and 70.6 ± 0.4 mN m^–1^ irradiation blank), indicating the presence of surfactants in the organic extracts, as well as the samples prior to extraction. This reduction in surface tension must be attributed to surfactants within the organic extracts, some of which are identified as lipids, discussed herein. Additionally, Section 3.5 details the surface tension of specific samples throughout the diel cycle.

These minimum surface tension ranges are higher than those observed in a previous study with 27.86–29.61 mN m^–1^ (mean of 28.5 ± 0.8 mN m^–1^) for the SML and 32.28–45.41 mN m^–1^ (mean of 35.7 ± 6.5 mN m^–1^) for the SSW from the Delaware Bay.^61^ However, the water from the Delaware Bay may be influenced by terrestrial runoff or anthropogenic surfactants such as linear alkylbenzenesulfonates and perfluoroalkyl substances,^104,105^ which could lead to lower surface tension minimums.^61^ Additionally, recent work has shown that nanoplastics can accumulate in the SML and disrupt lipid and surfactant films in the SML, which may be more prevalent in ocean regions with elevated anthropogenic inputs.^106^

### 3.2 Individual Lipid Classes Identified in the SML and SSW Samples

The percent contribution of individual lipid classes to the total lipid pool differs in the SML compared to the SSW throughout the day, as shown in Figure 1 (and Table S4). Figure 1d,h shows variability in the total fractions of specific lipid classes when comparing the SML and SSW compositions for each of the samples throughout the day, with the most noticeable difference in the Afternoon when the SML and SSW samples consistently showed differences in lipid abundances. The differing lipid compositions demonstrate that the SML and SSW are distinct microenvironments. Previous work from Triesch et al.^16^ shows distinct lipid compositions between the SML and SSW, supporting this observation. Additionally, a mesocosm study showed the SML to be a biogeochemical reactor for autochthonous organic matter,^14^ supporting the SML as a unique microenvironment. This indicates that the lipids in the SML are not directly transported from the SSW at the same ratios, since specific lipids undergo preferential transportation, or it may indicate that selective lipid production or loss mechanisms in the SML lead to differences in the compositions.

**Figure 1:**
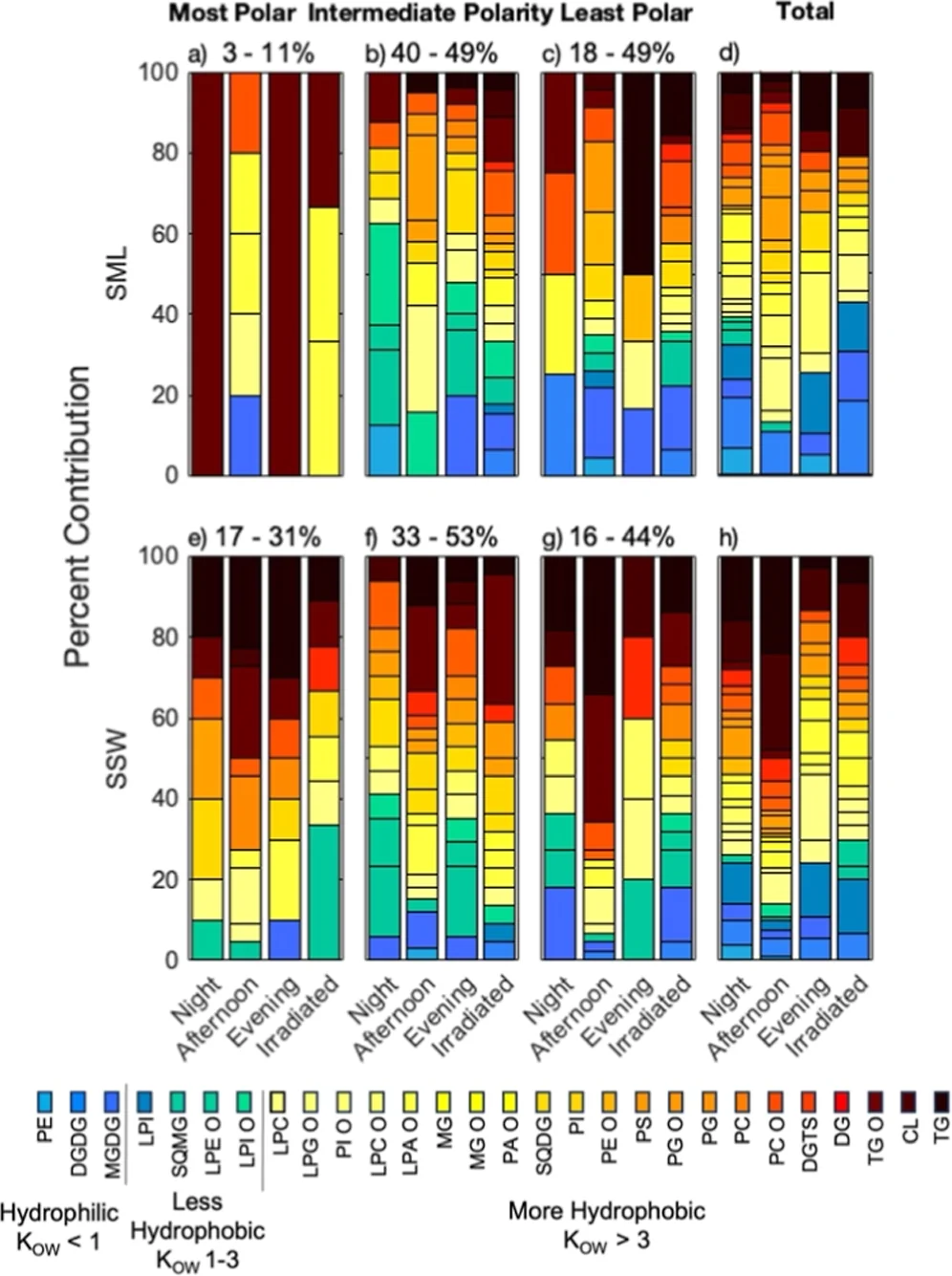
Fraction of the number of lipids in a given class out of the total lipids identified in the SML (a–d) and SSW (e–h) for each polarity fraction. Percentages represent the range of fractions of lipids identified in each polarity region, across the four samples in the SML and SSW. Lipid classes are ordered and colored by the octanol–water partition coefficient (*K*_OW_)^100^ and separated into hydrophilic (*K*_OW_ < 1), less hydrophobic (*K*_OW_ of 1–3), and more hydrophobic (*K*_OW_ > 3).

MGDG has the largest fraction in the SML, particularly in the Night and Irradiated samples (12% and 18% respectively; Figure 1d and Table S4), while the fraction in the SSW is lower (6% and 7%, respectively; Figure 1h and Table S4). MGDG, one of the most common glycolipids in plankton,^22,25^ has a sugar-containing headgroup expected to increase its solubility, thereby limiting its surface-activity, transport with bubbles, and enrichment in the SML,^107^ relative to lipid classes with higher surface activity, such as lysophospholipids. For example, the critical micelle concentration, the concentration at which surfactants start to form micelles, is higher for glycolipids like MGDG (6.3–13.1 μM)^108^ than lysophospholipids in general (0.007–4.4 μM),^103^ further demonstrating that lysophospholipids are generally more surface-active than glycolipids like MGDG. Its higher abundance in the SML suggests that MGDG may have higher relative production rates in the SML.

TG, by contrast, is a nonpolar molecule with a surfactant-like structure that is attracted to interfaces like the SML. The TG lipid class shows on average a higher fraction in the SSW compared to the SML, with the highest abundance in the SSW Afternoon sample (24%; Figure 1h and Table S4) and a relative depletion in the SML Afternoon sample (3%; Figure 1d and Table S4). The high abundance of TG in the SSW runs counter to the idea that compounds with more amphiphilic-like structures should be preferentially transported with bubbles to the SML. Instead, these results suggest that TGs, common storage molecules in phytoplankton,^34,47^ have a higher relative production rate in the SSW compared to the SML.

Of the lipid classes identified, PE has the lowest *K*_ow_, indicating that it is the most hydrophilic. Here, there is a larger fraction of PE in the SML (10% in the Evening; Figure 1d) compared to the SSW (3% in the Evening; Figure 1h). Similarly observed by Triesch et al.,^16^ there was a higher percent composition of PE in the SML compared to the SSW. This further demonstrates that there is possible production of PE and other soluble, hydrophilic lipids in the SML since they were likely not preferentially transported on bubbles.

The percent contribution of individual lipid classes to the total lipid pool also differs in the three polarity fractions in both the SML (Figure 1a–c) and the SSW (Figure 1e–g) throughout the day. Figure 1 shows the increase in the fraction of more hydrophobic lipid classes identified in both the SML and SSW as the polarity moves from more polar to least polar, in both the SML and SSW, as expected. The most identifications were with the intermediate and least polar retention times, in both the SML and SSW (Figure 1). The Intermediate Polarity fraction has the most diversity of lipid classes in both the SML and SSW (Figure 1), while the least diversity is in the Most Polar fraction, especially for the SML (Figure 1), which is likely due to the small number of lipid identifications. Radoman et al.^62^ found higher abundance of compounds in the Intermediate Polarity fraction and least abundance in the Lower Polarity fraction in both their SML and SSW samples, somewhat consistent with this work. Within the Intermediate Polarity fraction, there is a larger fraction of Hydrophilic and Less Hydrophobic lipid classes in the SML than in the SSW (Figure 1b,f). The Least Polar fraction shows more general similarity between the magnitude of the Hydrophilic and Hydrophobic fractions in the SML and SSW (Figure 1c,g). Overall, the SML has a larger fraction of Hydrophilic lipid classes than the SSW across polarity fractions (Figure 1), which is unexpected but may further support that some lipids may be independently produced in the SML rather than directly transported from the SSW.

### 3.3 Differences and Overlap in Specific Lipids Identified in the SML and SSW

Of the total lipids identified, only 33 were found in both the SML and SSW samples, as discussed later. This means 90% of the lipids identified are unique to the SML or the SSW and suggests little exchange between the layers. Some previous work shows transport between the SML and SSW^13^ which may not be as prevalent in this work, as discussed later. Because there is only ∼10% overlap in the lipids, these two layers can be considered distinct, with different organic sources and sinks. There are 130 lipids that were identified in only the SML (Figure 2a) and 156 lipids that were identified in only the SSW (Figure 2b). Of those lipids only identified in the SML, 62 are phospholipids and 22 are glycolipids (Figure 2a). Of those lipids only found in the SSW, 61 are phospholipids and 23 are glycolipids (Figure 2b). The remaining 25 identified in only the SML and 72 identified in only the SSW are classified as “Other Lipids” (listed in Table 1 and shown in Figure 2c). The ratios of hydrogen to carbon (H/C) and oxygen to carbon (O/C) vary for the identified lipids but follow the same general pattern of high H/C (mean of 1.67 ± 0.20) and low O/C (mean of 0.33 ± 0.16) characteristic of lipids (Figure 2). For comparison, Figure S2 shows the H/C and O/C ratios for lipids identified in the blanks of both the SML and SSW, which were excluded from this analysis.

**Figure 2:**
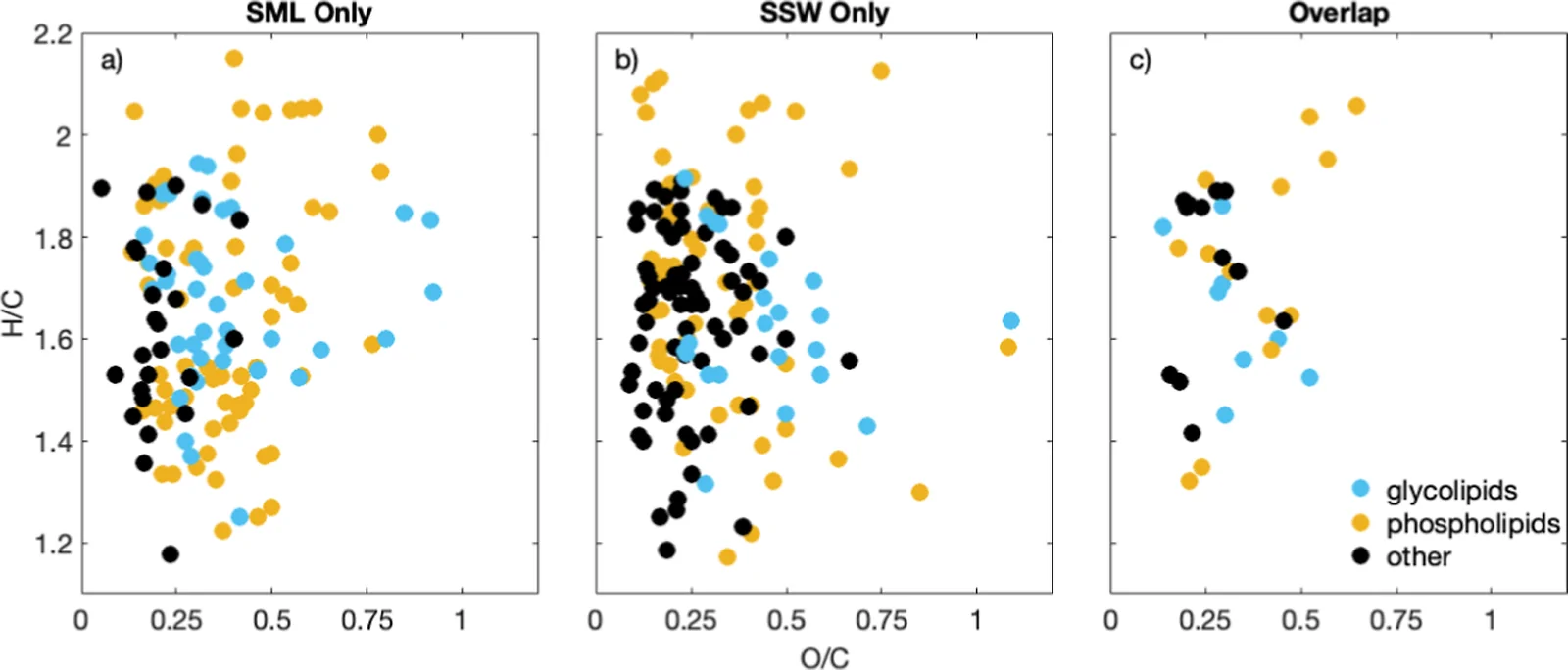
van Krevelen diagrams of the H/C and O/C ratios for lipids identified in the SML only (a), SSW only (b), and in both the SML and SSW (c), colored by the lipid type. The specific lipids in (c) are listed in Table S5.

For the lipids identified only in the SML, the H/C values are 1.18–2.15 (mean of 1.65 ± 0.21), the O/C values are 0.05–0.92 (mean of 0.35 ± 0.18), and the nitrogen to carbon (N/C) values are 0–0.11 (mean of 0.006 ± 0.015) (Figure 2a). The elemental ratios of the lipids only identified in the SSW are similar, with H/C of 1.17–2.27 (mean of 1.67 ± 0.20), O/C of 0.09–1.10 (mean of 0.30 ± 0.17), and N/C of 0–0.07 (mean of 0.01 ± 0.02) (Figure 2b). The preferential enrichment of certain lipid classes in the SML may be due to molecular properties, indicated by H/C and O/C.^15,62,109^ However, if high H/C was the main factor in the preferential transport of specific lipid classes from the SSW to the SML, we would expect a higher H/C and lower O/C for lipids only observed in the SML. Instead, we observe similar H/C and O/C in both and a difference in the glycolipid abundance compared to the phospholipid abundance. This in turn may indicate that the SML and SSW are each unique environments with different lipid production and degradation mechanisms or that high H/C is not the only contributor for enrichment in the SML. Previous work showing a preferential enrichment of high H/C in the SML used different methods for the SML collection but similar methods for the organic extractions, with PPL cartridges,^62^ which retain similar compounds as C18 SPE cartridges.^79^

Lipids that are identified in both the SML and the SSW are more likely to be transported between the two, but they may also be independently produced and/or degraded in both. The properties of the lipids identified in both may help inform the sources and properties of the other lipids and surfactants in the SML. There are 33 lipids (13 phospholipids, 8 glycolipids, and 12 “Other Lipids” (Table 1 and Figure 2c) that were identified in all of the samples (∼10% of the total number of lipids identified). While transport between the layers may occur, the low overlap between the SML and SSW lipid identifications may reflect that each is a unique environment with different lipid sources and sinks. Recent work has shown differences in the photochemistry of the SML and SSW during a phytoplankton bloom, suggesting that the two may have different sources and sinks.^110^ Differences in environmental conditions during sampling, especially within individual time points, from SML to SSW, were very minor or nonexistent (see Table S1) and thus do not contribute to the small overlap in the number of lipids identified in both the SML and SSW.

The H/C values for the ubiquitous lipids are 1.32–2.06 (mean of 1.72 ± 0.19), and the O/C values are 0.14–0.65 (mean of 0.33 ± 0.13) (Figure 2c). The N/C values are 0–0.06 (mean of 0.001 ± 0.006). Previous work has marked formulas with H/C greater than 1.32, O/C less than 0.6, and N/C less than 0.126 to be lipid-like when identifying complex matrices with high resolution MS,^111^ consistent with most lipids identified in both the SML and SSW here. While the H/C values are similar for lipids measured in only the SML (1.65 ± 0.21) and in only the SSW (1.67 ± 0.20), the H/C values of the 33 lipids that were ubiquitous in all the samples are slightly higher (1.72 ± 0.19). These higher H/C values indicate more hydrophobic molecules attracted to bubble surfaces, making them more likely to be transported from the SSW to the SML.^61^ One might expect the most amphiphilic, surface-active molecules to have a lower O/C as well. However, that is not observed here since the O/C value for the ubiquitous lipids (0.33 ± 0.13) is similar to that of the SML only and SSW only lipids (0.35 ± 0.18 and 0.30 ± 0.17, respectively).

The five lipids observed both in the SML and SSW with the highest intensity are phospholipids PC O 9:4 and LPI 14:7, triacylglycerols TG 25:0 and TG 30:6, and alkyldiacylglyceride TG O 21:6 (Figure 2c and Table S5). These five have H/C of 1.35–1.86, O/C of 0.18–0.41, and N/C of 0–0.06 (Figure 2c and Table S5). LPI is observed in bacterial membranes and can indicate degradation processes.^112^ TGs are typically energy storage molecules in eukaryotic phytoplankton.^47^ Thus, both bacterial and phytoplankton sources contribute to the lipid pool observed in both the SML and SSW. TG has properties that would support its transport from the SSW to interfaces such as the SML. This supports the idea that the lipids present in both the SSW and SML could be those transported between the SSW and the SML and may explain why TG has such high intensity in the overlapping lipid pool.

An EF of 1.06 was calculated for the lipids identified in both the SML and SSW for the samples in the Night, Afternoon, and Evening. Of the lipids identified in both the SML and SSW, higher intensities were observed overall in the SML compared to the SSW. This enrichment is consistent with the accumulation of surface-active lipids, such as TG, in the SML. This was also previously observed by Triesch et al.,^16^ where targeted lipid analysis using Iatroscan thin layer chromatography and flame ionization for the detection of organics in DOM had a lipid EF of 1.1–1.4 (mean of 1.3).

When comparing all lipids identified across both layers, individually and together, there are also enrichments (EF > 1) for all glycolipids, except SQDG (median EF of 0.34), and an enrichment of 7 out of the 18 identified phospholipids (Figure 3 and Table S4). This may be due to glycolipids generally being more surface-active than phospholipids, but the specific surfactant strength varies.^103,108^ The preferential enrichment of these lipids in the SML could be derived from their transport from the SSW to the SML, higher production within the SML, and/or higher rates of degradation in the SSW, as discussed in the following sections.

**Figure 3:**
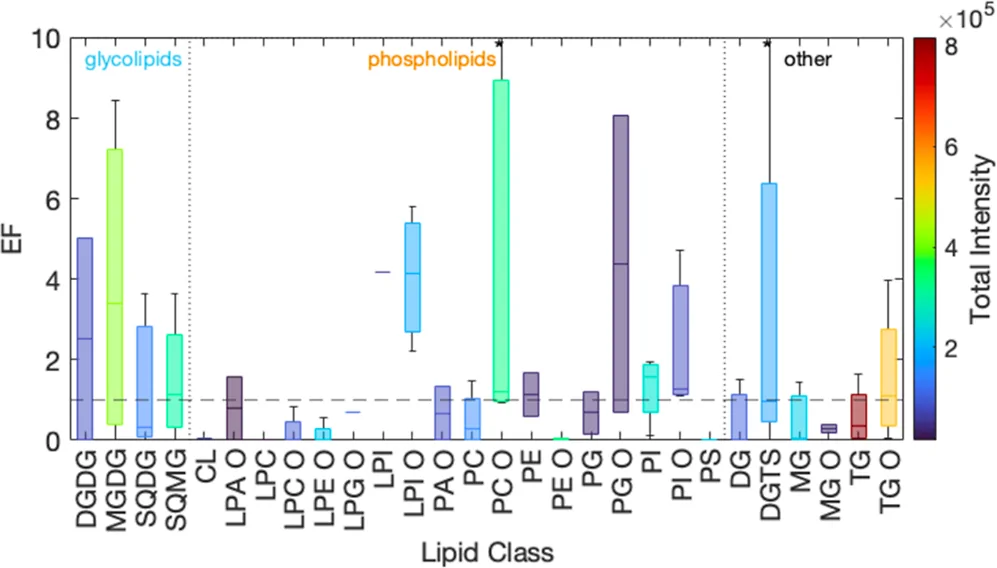
EF for the individual lipid classes identified. The color of each bar indicates the total summed intensity of lipids identified in the SML and SSW for each lipid class, for all four sample times. The dashed line represents an EF of 1. The horizontal lines represent the median values, and the boxes range from the lower to upper quartiles, where available. Error bars extend to the highest and lowest points, and no outliers were observed. The asterisks indicate error bars that exceed the bounds of the figure (PC O extends to an EF of 16.5 and DGTS extends to an EF of 11.8).

### 3.4 Lipid Sources in the SML and SSW

Intact lipids are used as biomarkers for living organisms since their polar headgroups are often lost quickly when they are no longer inside living cells.^36–40^ Here, we discuss the presence and EF of specific lipids and lipid classes that are used as biomarkers and tracers for bacteria and phytoplankton with the goal of identifying sources of lipids and surfactants in the SML. Figure 3 shows the EF of different lipid classes for all of the lipids identified in the SML and/or SSW samples.

PE is a common phospholipid found in heterotrophic bacterial membranes and is used as a tracer of bacterial sources.^41,113^ Here, EF_PE_ is 1.14 (Figure 3 and Table S4), indicating that enhanced bacterial activity may be one source of lipids to the SML. Another lipid used as a biological tracer in seawater is the glycolipid DGDG, which is associated with cyanobacteria^114,115^ and is one of the most common glycolipids in plankton.^22,25^ Here, DGDG is enriched in the SML (EF_DGDG_ of 2.51) (Figure 3 and Table S4).

Some phospholipids are unique to specific organisms, such as PG, which is used as a biomarker for phytoplankton.^34^ EF_PG_ is 0.68 (Figure 3 and Table S4), suggesting that there is no phytoplankton enrichment in the SML and that the phytoplankton likely remains in the SSW over the course of this study. The ratio of PE (bacteria tracer) to PG (phytoplankton tracer) or PE/PG is used to determine the sources of lipids.^42^ Here, there is a larger PE/PG in the SML (2.48) and smaller PE/PG in the SSW (0.55) in all of the samples combined (Table 2), supporting the presence of more bacteria, including cyanobacteria, in the SML and more phytoplankton in the SSW. Previous work has also shown higher PE/PG in the SML than in the SSW.^16^ Cyanobacteria are enriched in the SML as demonstrated by the higher PE/PG in the SML, indicating that the previously discussed glycolipids enriched in the SML are likely coming from a bacterial source.

**Table 2:** Average Lipid Ratios and Lipolysis Index for the SML and Associated SSW

	PE/PG	SQDG/PG	DGTS/PC	lipolysis index
SML	2.48	7.96	2.11	0.18
SSW	0.55	1.64	0.62	0.11

TG was significantly depleted in the SML compared to the SSW, as shown with an EF_TG_ of 0.37 (Figure 3 and Table S4). This lack of enrichment is consistent with the lack of enrichment of PG in the SML since both TG and PG are linked to phytoplankton production.^47^ The depletions of TG and PG in the SML relative to the SSW further indicate that phytoplankton are more important sources of lipids in the SSW than the SML. TGs are also labile after cell lysis in the ocean^116^ and could be depleted due to the increased cell degradation in the SML.^117^

The linkage between the fatty acid chains and the polar headgroup can also be unique to certain organisms. For example, ether-linked lipids are primarily from archaea and some bacteria.^28^ Table 1 identifies the lipids discussed in this paper to contain either ether or ester linkages. Here, the ether-linked lipids had an average EF of 0.51. The lack of enrichment in the SML for all ether-linked lipids together could indicate that archaeal lipids are not preferentially produced in or transported to the SML. This aligns with previous studies reporting that some archaea are found at lower abundance in surface waters but increase in abundance with increasing depth.^118^

Surface waters have nutrient deficiencies,^52^ and organisms can adapt to these conditions by producing glycolipids instead of phospholipids.^34,53,54^ In the SML, the glycolipids listed in Table 1 were enriched (median EF of 3.27), while the phospholipids listed in Table 1 were not (median EF of 0.56), which indicates that the SML may be nutrient-limited compared to the SSW.

The ratio of SQDG to PG (SQDG/PG) was 7.96 in the SML and 1.64 in the SSW in all of the samples combined (Table 2). The higher SQDG/PG in the SML may indicate sources from photosynthetic microorganisms and nutrient limitation.^56^ Cyanobacteria can substitute PG for SQDG under phosphorus-limited conditions.^56^ Therefore, this is consistent with cyanobacteria as a possible dominant source contributing to the lipid pool in the SML. Another ratio that is linked to nutrient availability is the ratio of betaine lipids to PC (DGTS/PC). The DGTS/PC was 2.11 in the SML and 0.62 in the SSW in all the samples combined (Table 2). When nutrients, such as phosphate, are limited, phytoplankton adapt by producing betaine lipids instead of PC.^58,59^ Larger DGTS/PC in the SML compared to the SSW further supports lower availability of nutrients in the SML.^56^

Other molecules, such as those containing polyunsaturated aldehydes, are also often related to nutrients in the seawater. In this study, the EF value was 0.96 overall for polyunsaturated aldehydes, showing a slight depletion in the SML. This could be attributed to a lack of available nutrients present in the SML, since that would slow the synthesis of polyunsaturated aldehydes.^34,46,119^

Together, the EF and ratios of specific lipids and lipid classes support enrichment of bacteria, specifically cyanobacteria, in the SML and enrichment of phytoplankton in the SSW. An apparent lack of nutrients present in the SML also contributes to the enrichment of glycolipids instead of phospholipids in the SML. Due to the limited number of samples, on 1 day, a clear diel trend with any of the EFs or lipid ratios cannot be observed.

### 3.5 Lipid Sinks in the SML

In addition to differential production rates of lipids in the SML and SSW and the preferential transport of specific lipids to the SML, degradation within the SML or SSW could contribute to lipid depletions (or enrichments) in the SML compared to the SSW.^120^ Degradation can either be due to enhanced (or reduced) rates of abiotic (e.g., via photochemical reactions)^121,122^ or biotic (e.g., breakdown by heterotrophic bacteria) processes.^123^ Photochemical degradation decreased the concentration of the glycolipid sterol^16^ by half in ∼3 h in the upper euphotic layer of the ocean,^124^ on a clear summer day in midlatitude waters.^125^ Harvey et al.^126^ showed that under anoxic and oxic conditions, cyanobacterial lipids are degraded microbially in 160 and 77 days, respectively. Some lipids in these samples show indicators of potential degradation, as demonstrated by the differences in chain length, EF, and lipid ratios for the SML and SSW samples collected at different points throughout the day, as well as for the Irradiated samples.

To investigate the effect of exposure to solar radiation on the fatty acid chain lengths of the identified lipids, the four SML samples (Afternoon, Evening, Night, and Irradiated) were evaluated and compared. Text S1 discusses and Figure S3 shows the number of carbons per lipid, separated by lipid class. The average carbon numbers of the identified lipids (Table 1) are lower for the Irradiated SML (18.2 ± 9.5) than in the Night SML (24.5 ± 9.6), reflecting the possible degradation of the fatty acid chains in the lipids due to solar radiation. A similar trend is observed with the number of carbons in the identified lipids from the Night SML (24.5 ± 9.6) compared to the Afternoon SML (21.3 ± 12.2) and Evening SML (16.3 ± 8.5). These samples have progressively more exposure to solar radiation and progressively smaller average carbon numbers. Previous studies have also shown that smaller fatty acid chains are linked to biological processes in the ocean, such as microbial growth, which could contribute to lower average carbon numbers in identified lipids throughout the day.^127,128^ This can also be seen in the percent contribution of PE, a biomarker for bacteria, in the SML which increases from the Night (0%; Figure 1d and Table S4) to the Afternoon (3%; Figure 1d and Table S4) and Evening sampling times (10%; Figure 1d and Table S4). However, the number of carbons per lipid identified from the Irradiated (18.2 ± 9.5) and Evening (16.3 ± 8.5) samples are the most similar, and since 0% of PE (Figure 1d and Table S4) was seen in the Irradiated samples, this further indicates the impact of solar radiation on the photochemical degradation of these lipids.

The average EF for the total lipid pool overall was 3.33 ± 4.13 for the Night, 0.79 ± 1.76 for the Afternoon, 0.92 ± 1.61 for the Evening, and 1.09 ± 1.48 for the Irradiated samples. Similar to the number of carbons, the EF decreases with enhanced solar radiation. The Afternoon, Evening, and Irradiated samples have lipids with similar, low total EF, compared to the EF of the Night, which suggests that irradiation breaks down lipids in the SML. However, there is a lot of variability between the lipid classes, as well as the individual samples, which suggests that any relationship of total lipid EF with light is complex. There could be production processes, such as primary production, but also degradation processes (i.e., photochemical or biological), accounting for this decrease in EF compared to the Night.

The average lipid EF (1.09 ± 1.48) for the Irradiation samples, which is greatly reduced from the Night (3.33 ± 4.13), is consistent with higher rates of lipid photochemical degradation in the SML with exposure to solar radiation. This trend in EF was similar, with the Night (3.33 ± 4.13) compared to the Evening (0.92 ± 1.61) as well, since the Evening had the most exposure to solar radiation throughout the day.

In addition to the differences in lipid composition across the four sample times, the surface tensions also differed. The surface tension minimums were higher in the Irradiated samples (58.2 mN m^–1^ for the SML; 56.6 mN m^–1^ for the SSW) than in the Night (33.5 mN m^–1^ for the SML; 48.1 mN m^–1^ for the SSW), indicating a loss of surfactants and/or a decrease in surface activity of these organics, due to irradiation. Photochemical reactions may cleave bonds, making the organics less surface-active. While lipids are not the only surfactants in the SML and SSW, the increase in surface tension and associated decrease in surface activity with irradiation is consistent with the decrease in lipid enrichment and the shorter fatty acid chain lengths of the lipids in the SML with irradiation. Previously, studies have shown this trend of decreased surface activity with decreased surfactant chain length in solutions of polyacrylamide sulfonate/alkyltrimethylammonium bromides and polydiallyldimethylammonium chloride/sodium alkylsulfate/sodium chloride.^23,24^ Biotic degradation of the lipids due to the bacterial activity in the SML could be occurring;^16^ however, abiotic degradation processes are more likely. The increased exposure to solar radiation and the decreased EF seen in the Irradiation samples, as well as the lack of PE (bacterial biomarker; Figure 1d and Table S3), support the idea that most of the degradation is due to photochemistry and not biotic processes. Lipids can be directly released from phytoplankton,^33^ which would cause a large increase in the lipids in the SSW with irradiation. While these observations highlight only one diel cycle, future work aims to confirm these trends across multiple days.

Free fatty acids are degradation products of lipids in general, while lysophospholipids are degradation products of phospholipids specifically and are used as tracers of degradation in the SML and SSW.^35,36,44^ The free fatty acids had a median EF of 1.82, and the lysophospholipids had an EF of 1.10 over all of the samples. Both classes of degradation products were enriched in the SML, which indicates higher overall degradation there compared to the SSW. We expect higher abiotic degradation in the SML compared to the SSW because the SML has more direct exposure to solar radiation.^117,129^ Previous work showed higher photochemical activity in the SML compared to SSW, during an induced phytoplankton bloom.^110^ Additionally, the results in Section 3.4 suggest a higher bacterial activity in the SML, which could lead to more biotic degradation in the SML; however, as discussed previously, abiotic processes are more likely to be the cause of degradation.

The potential higher degradation in the SML compared to the SSW is also supported by the Lipolysis Index.^35,44^ The Lipolysis Index of dissolved lipids ranges from 0.13 to 0.53, indicating the presence of lipolysis.^16^ Averaged over all of the samples, the Lipolysis Index of the SML in this study is greater (0.18) than that of the SSW (0.11) (Table 2), indicating more degradation in the SML. A higher Lipolysis Index in the SML was measured in previous studies as well.^16,21,34^ In addition to being products of lipolysis, DG and MG can also be assimilated by microorganisms.^16^ They were both depleted in the SML (EF_DG_ of 0 and EF_MG_ of 0.06; Figure 3 and Table S3). Because of the higher bacterial activity in the SML compared to the SSW, these metabolites could be taken up more readily in the SML than in the SSW, thus leading to the lack of enrichment there.

Overall, the SML has indicators of more degradation than the SSW. While some may be biotic, photochemical degradation plays a large role in the composition of lipids and surfactants in the SML, resulting in lower lipid carbon chain lengths with exposure to solar radiation.

## 4 Conclusions

Several lipid classes were identified in the SML and SSW collected at multiple time points throughout the day in the North Atlantic Ocean. We identified 28 classes of lipids overall and 163 individual lipids in the SML and 189 in the SSW, and the lipids identified in the two layers had only ∼10% overlap. We observed that the total intensity of lipids identified in both the SML and SSW showed an enrichment in the SML, compared to the SSW (average EF of 1.68 ± 1.43 for the Night, Afternoon, and Evening samples). The observed enrichments may be due to different processes, such as production or destruction mechanisms in the SML and SSW and/or overall transport of total lipids between the SSW and SML.

Taken together, this work shows that (i) the SML is likely a unique microenvironment compared to the SSW at this location and date; (ii) bacteria may be producing more lipids in the SML than phytoplankton; (iii) there is possible nutrient depletion in the SML; and (iv) there is more potential lipid degradation in the SML compared to the SSW. The enrichment of PE in the SML (EF_PE_ of 1.14) and larger PE/PG in the SML (2.48) compared to the SSW (0.55) indicate that bacteria may be an important source of lipids to the SML. However, the lower availability of nutrients in the SML could cause a decrease in phospholipid production in both photosynthetic bacteria and phytoplankton. There was an enrichment of glycolipids (EF_GL_ of 3.27) and not phospholipids (EF_PL_ of 0.56) in the SML, as well as larger DGTS/PC and SQDG/PG in the SML (2.11 and 7.96, respectively) compared with the SSW (0.62 and 1.64, respectively). Based on these nutrient depletion biomarkers, phytoplankton could also be a source of lipids to the SML. Increased lipid degradation likely occurs in the SML, compared to the SSW, as demonstrated by the enrichment of free fatty acids (EF of 1.82) and lysophospholipids (EF of 1.10) and a larger Lipolysis Index in the SML (0.18) than that in the SSW (0.11).

These results further our understanding of lipids and associated compounds in the surface ocean, their preferential enrichment in the SML, and the potential processes influencing their composition. This work highlights the complexity of the lipid pool in the SML and SSW, which may be further represented in air–sea exchange models. Because of the preferential enrichment of some lipids, as well as the differences in the lipids in the SML and SSW, the lipids in the SML cannot be assumed to be directly correlated with those in the SSW, and the SML should be considered its own microenvironment. While the data presented here provide important insights, the sample sizes are small, so strong conclusions related to diel/seasonal trends or spatial representativity cannot be made and will need to be tested with future work.

Additionally, DOM can originate from multiple sources; therefore, it is important to understand its properties in all regions of the ocean including the coasts. The hydrographic region in which samples are collected can vary in primary productivity and biological communities present, thus affecting the molecular composition of DOM. Coastal regions may have additional inputs, such as from terrestrial and anthropogenic sources, and contain DOM not observed in other hydrographic regions. This work shows that lipids can be used to understand surfactants and biological processes at the air–sea interface. However, more work can be done to associate these lipids and surfactants in different hydrographic regions and times of year to advance our understanding of the surface ocean and air–sea exchange processes. Additionally, future work should investigate the impact of these surfactant lipids on air–sea gas exchange rates and the production rates and physicochemical properties of sea spray aerosol particles.

## Supplementary Material



## Notes

Table of environmental parameters during sampling (Table S1); figure showing the setup of irradiation experiments (Figure S1); table describing the gradient elution and polarity groups for LC (Table S2); table of *K*_OW_ of lipid classes (Table S3); table of mean carbon numbers, fraction of total lipids in the SML and SSW, median EF, and total intensity for each lipid (Table S4); figure showing the O/C and H/C in the blanks (Figure S2); table of H/C, O/C, and intensities of lipids in both the SML and SSW (Table S5); text describing the carbon numbers of individual lipid classes (Text S1); and figure showing the carbon number in each lipid class (Figure S3) (PDF)
