# Supplementary material for: Distinct Lipid Compositions in Seawater and the Sea Surface Microlayer Measured with Liquid Chromatography and High-Resolution Mass Spectrometry
Source: ACS Earth Space Chem. 2026 Jul 30;10(8):1870–83. doi: 10.1021/acsearthspacechem.6c00084 (PMC13501954; doi:10.1021/acsearthspacechem.6c00084)
Supplement: Supplementary file 1 [file sp6c00084_si_001.pdf]

Supporting Information for

Distinct lipid compositions in seawater and the sea surface microlayer measured with liquid chromatography and high-resolution mass spectrometry

Amber E. Birt[1], Daniel Ammer[1], Felix E. Agblemanyo[2], Andrew S. Wozniak[2], Amanda A. Frossard[1,*]

1. Department of Chemistry, University of Georgia, Athens, GA, 30606, USA
2. School of Marine Science and Policy, University of Delaware, Lewes, DE, 19958, USA

*Corresponding author: Amanda A. Frossard, afrossard@uga.edu

Number of pages: 11

Number of texts: 1

Number of tables: 5

Number of figures: 3

Table S1. Seawater and atmospheric conditions during the sampling times, reported previously by *Agblemanyo et al.*[1].

		Sampling Times	Num. Dips	Chl-a Conc. (μg L^{-1})	Sea Surface Temp. (°C)	Wind Speed (m s^{-1})
Night*	SML	2:39 – 5:04	63	0.15	26.2 ± 0.04	6.1 ± 0.9
	SSW	05:10	--	0.16	26.2 ± 0.003	4.5 ± 0.5
Afternoon	SML	13:00 – 13:55	30	0.22	26.0 ± 0.05	3.3 ± 0.5
	SSW	14:10	--	0.22	26.1 ± 0.003	3.6 ± 0.4
Evening	SML	18:34 – 20:06	38	0.19	26.6 ± 0.1	4.0 ± 0.8
	SSW	20:21	--	0.21	26.4 ± 0.01	4.2 ± 0.2

*The Irradiated sample was collected with the Night sample, so all of the conditions are the same

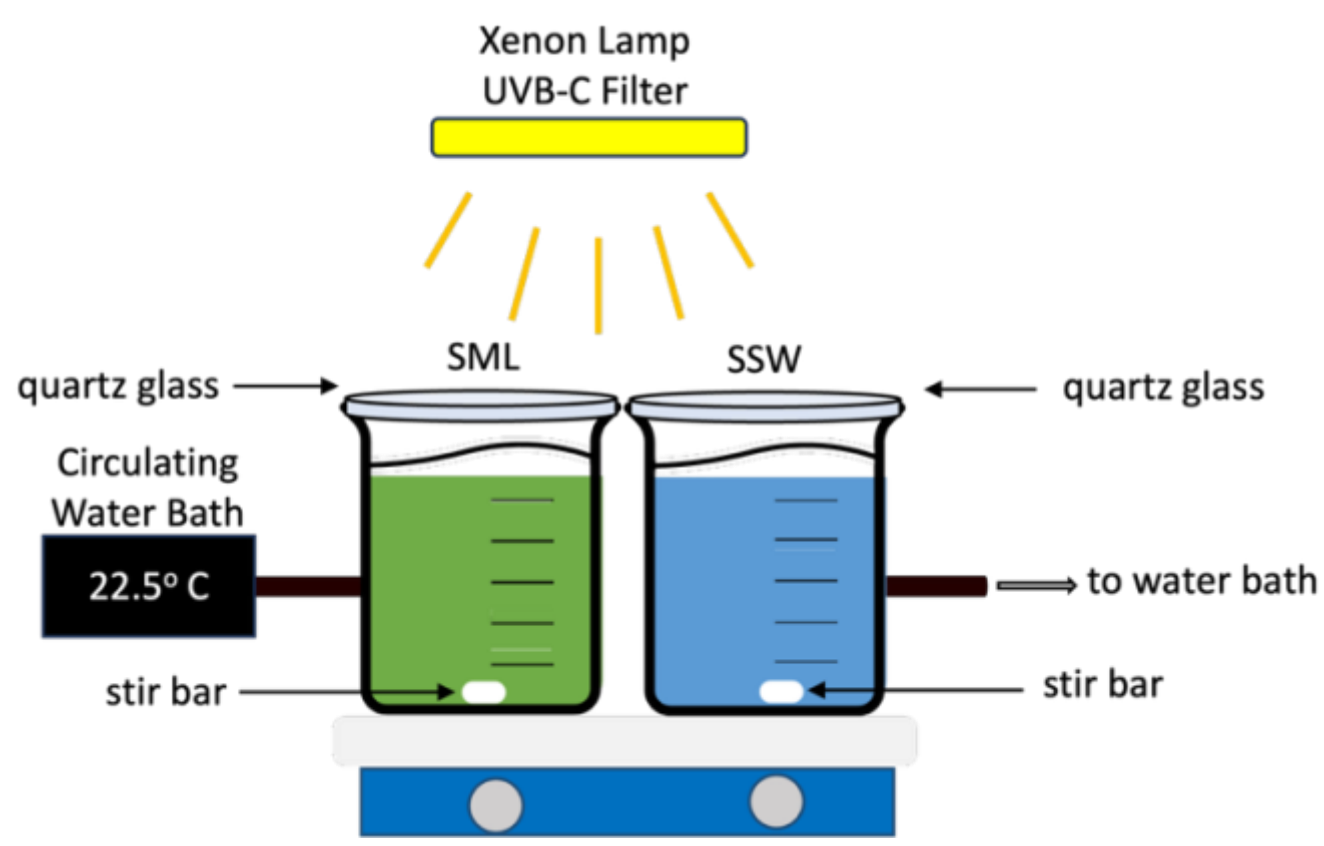

Figure S1. Schematic illustrating the irradiation set up, described in Section 2.2. This includes the 400 mL jacketed beakers with the SML and SSW samples and the quartz glass lids, as well as the circulating water bath, flowing through the jacketed beakers.

Table S2. Fraction of acetonitrile (ACN %) in the gradient liquid chromatography separation, in both ionization modes of the mass spectrometer, and the corresponding polarity type.

	**Negative Mode**		**Positive Mode**	
**Polarity Type**	**Time (minutes)**	**Fraction of ACN (%)**	**Time (minutes)**	**Fraction of ACN (%)**
Most Polar	N/A	N/A	0-3	45-65
Intermediate Polarity	0-7	65-85	3-7	65-85
Least Polar	7-12	85-100	7-12	85-100

Table S3. Lipid classes identified in this study, ordered and colored by the octanol-water partition coefficient (K_{OW})[2] as shown in Figure 1.

Acronym	Color	K_{OW}
**Hydrophilic**		**< 1**
PE		-4.4
DGDG		-3.8
MGDG		-3.5
**Less Hydrophobic**		**1-3**
LPI		1.2
SQMG		2.6
LPE O		2.7
LPI O		3
**More Hydrophobic**		**> 3**
LPC		5.6
LPG O		5.7
PI O		5.8
LPC O		5.9
LPA O		6.5
MG		7.4
MG O		7.6
PA O		7.8
SQDG		10
PI		10.2
PE O		11.2
PS		12.1
PG O		12.2
PG		12.3
PC		12.9
PC O		14.4
DGTS		14.7
DG		16.2
TG O		22.8
CL		23.9
TG		25.2

Table S4. Fraction of total lipids in the SML and SSW, median EF and total intensities in the SML and SSW for each lipid, as shown in Figures 1, 3, and S1.

	Night		Irradiated		Afternoon		Evening			Intensity	
**Lipid Class**	Fraction in SML (%)	Fraction in SSW (%)	Fraction in SML (%)	Fraction in SSW (%)	Fraction in SML (%)	Fraction in SSW (%)	Fraction in SML (%)	Fraction in SSW (%)	Median EF	SML	SSW
**Glycolipids**											
DGDG	7	4	0	0	0	1	5	0	2.51	36019	9038
MGDG	12	6	18	7	11	4	0	5	3.39	156326	50024
SQDG	4	4	12	0	0	2	5	5	0.34	44836	31014
SQMG	9	10	12	13	0	2	15	14	1.12	93822	64043
**Phospholipids**											
CL	3	0	0	3	3	1	0	0	0.06	8015	36365
LPA O	2	2	0	7	0	0	0	0	0.79	7073	8427
LPC	1	0	0	0	0	3	0	0	0	7371	8927
LPC O	1	4	0	3	3	8	0	5	0.03	7766	45309
LPE O	0	2	3	3	0	1	5	16	0.03	8446	104628
LPG O	2	0	9	3	13	0	0	0	0.71	48198	12790
LPI	1	2	0	0	3	0	0	0	4.19	29410	4540
LPI O	6	4	6	3	8	0	20	3	4.16	83006	15177
PA O	3	2	0	0	5	0	0	3	0.67	31606	16360
PC	6	4	3	7	0	4	0	8	0.29	29989	48727
PC O	7	2	3	7	3	2	5	5	1.20	108110	58352
PE	0	0	0	0	3	1	10	3	1.14	13946	10289
PE O	1	0	3	3	5	0	0	3	0.03	16113	149270
PG	1	4	0	0	3	1	0	0	0.68	5634	18877
PG O	0	0	3	3	11	1	0	0	4.37	17403	5848
PI	4	8	3	3	8	3	5	5	1.56	50165	83989
PI O	2	2	3	0	3	1	5	3	1.25	32657	13003
PS	0	2	0	0	3	0	0	5	0	2019	110572
**Other Lipids**											
DG	3	4	0	3	0	3	0	0	0	20963	34758
DGTS	6	2	0	3	8	4	5	3	0.96	63264	30115
MG	2	4	0	7	3	5	0	0	0.06	38425	77446
MG O	1	2	0	0	3	2	5	0	0.30	6847	17600
TG	9	10	12	13	3	24	0	11	0.37	141133	674976
TG O	6	16	9	7	3	24	15	3	1.11	64276	201967

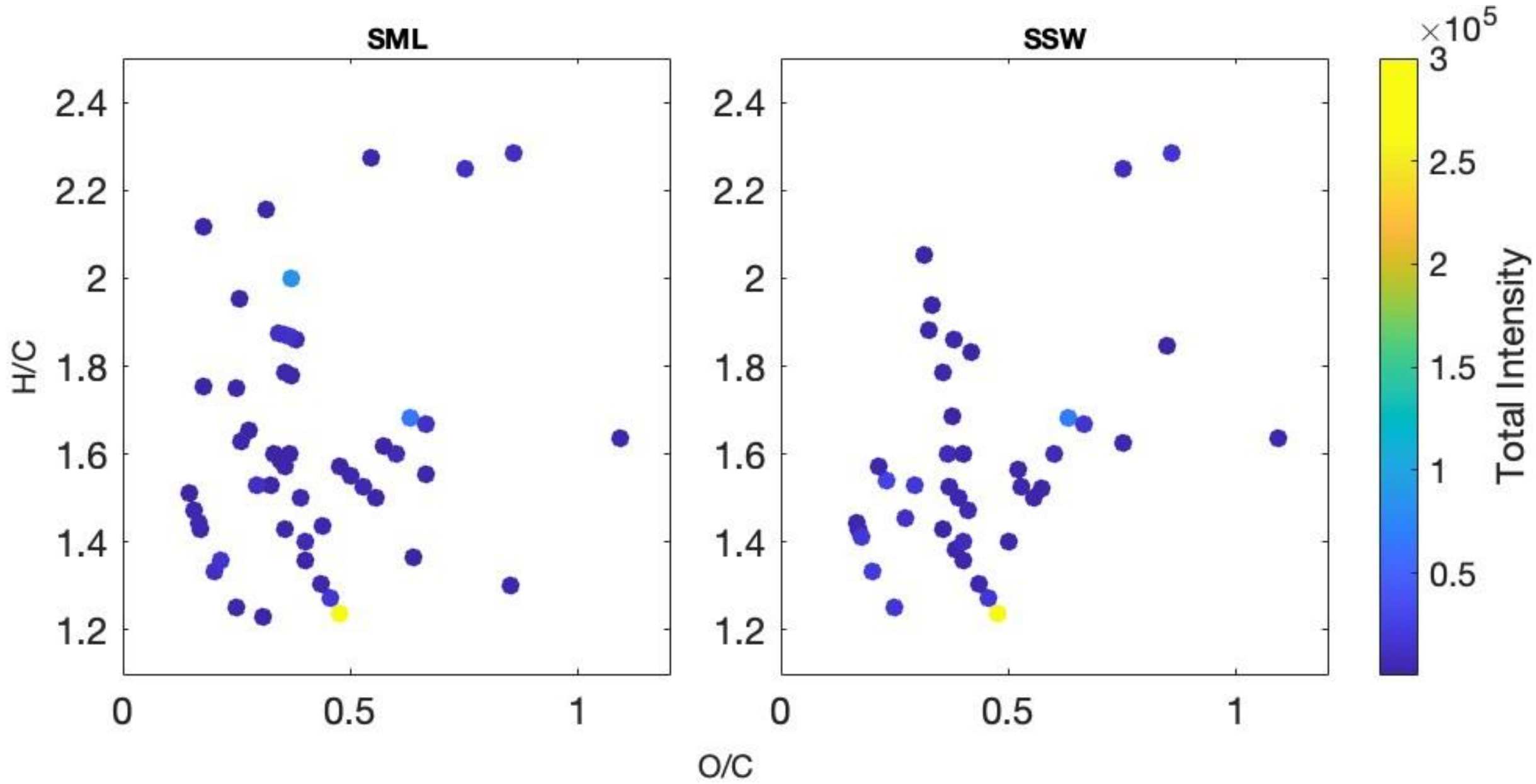

Figure S2. van Krevelen diagram of the H/C and O/C ratios for lipids identified in the blanks of both the SML and SSW. Points are colored by the intensity of the identified lipid. The sample intensities range from 1.2 x 10^3 - 3.1 x 10^5, and the blank intensities were all below 9.5 x10^4, except MGDG 12:6 which is around 2.9 x 10^5 (SML) and 2.6 x 10^5 (SSW). The lipids identified in the blanks were all removed from the sample identifications.

Table S5. H/C and O/C ratios for lipids identified in both the SML and SSW labeled by the specific lipid and ordered by intensity.

Name	H/C	O/C	Intensity
PC O-9:4	1.65	0.41	31930
TG 25:0	1.86	0.24	20943
TG 30:6	1.52	0.18	19023
LPI 14:7	1.35	0.24	19020
TG O-21:6	1.42	0.21	15440
LPI O-18:0	2.04	0.52	13250
SQDG 32:4	1.71	0.29	11210
MGDG 24:1	1.82	0.14	10030
MGDG 31:9	1.45	0.30	9726
SQMG 30:5	1.69	0.28	9377
PE 14:4	1.58	0.42	9287
DGDG 35:8	1.56	0.35	9075
DG 14:3	1.53	0.16	8724
PC O-37:6	1.78	0.18	8063
TG 28:0	1.87	0.19	7053
PC 37:2	1.91	0.25	7008
LPI O-8:0	2.06	0.65	6851
PI O-12:0	1.95	0.57	6367
DGTS 17:0	1.89	0.30	6128
TG O-8:1	1.64	0.45	5824
PI 32:4	1.73	0.32	5764
LPC O-35:7	1.77	0.26	5726
PC 9:3	1.65	0.47	5463
SQMG 16:4	1.60	0.44	5106
DGDG 28:0	1.86	0.29	4981
TG 26:0	1.86	0.21	4734
TG 22:1	1.76	0.29	4615
PI 20:0	1.90	0.45	4575
LPA O-22:10	1.32	0.21	4480
TG O-15:0	1.89	0.28	4107
SQMG 12:4	1.52	0.52	3903
DGTS 25:1	1.86	0.20	2614
TG O-12:1	1.73	0.33	1492

Text S1. Studies recommend separating lipids based on individual lipid classes, rather than comparing carbon numbers overall.[3] **Figure S3** shows all identified lipid classes and their respective average carbon number. Overall for the SML and SSW identifications combined, each lipid class has average carbon numbers between 12-32, with the overall average around 22 (**Figure S3**; **Table 1).** The lipid class with the highest average carbon number is monoalkylglcerophosphoethanolamine (LPE O, 31.9), while the lowest average carbon number is observed for monoalkylglycerophosphoinositol (LPI O, 13.2) (**Figure S3**; **Table 1)**. Previous work has measured the average carbon numbers for specific lipids in seawater, consistent with this work. For example, *van Mooy et al.*[3] observed average carbon numbers of 38 and 30 for PC and SQDG, respectively. These are higher than the average carbon numbers of 31 and 18 for PC and SQDG, respectively, observed here (**Figure S3**; **Table 1)**. The difference could be attributed to the seawater collected, since *van Mooy et al*.,[3] collected samples throughout the euphotic zone and at lower depths. At deeper depths there is less solar irradiation to potentially degrade the molecules, resulting in longer fatty acid chains.[4, 5] Additionally, samples in *van Mooy et al.,*[3] were extracted using the Bligh and Dyer method[6] as opposed to the solid phase extraction method used here. The liquid chromatography column and gradient methods were also different compared to what was used in this work. These factors could have also contributed to the discrepancies between the results from *van Mooy et al.,*[3] and this work.

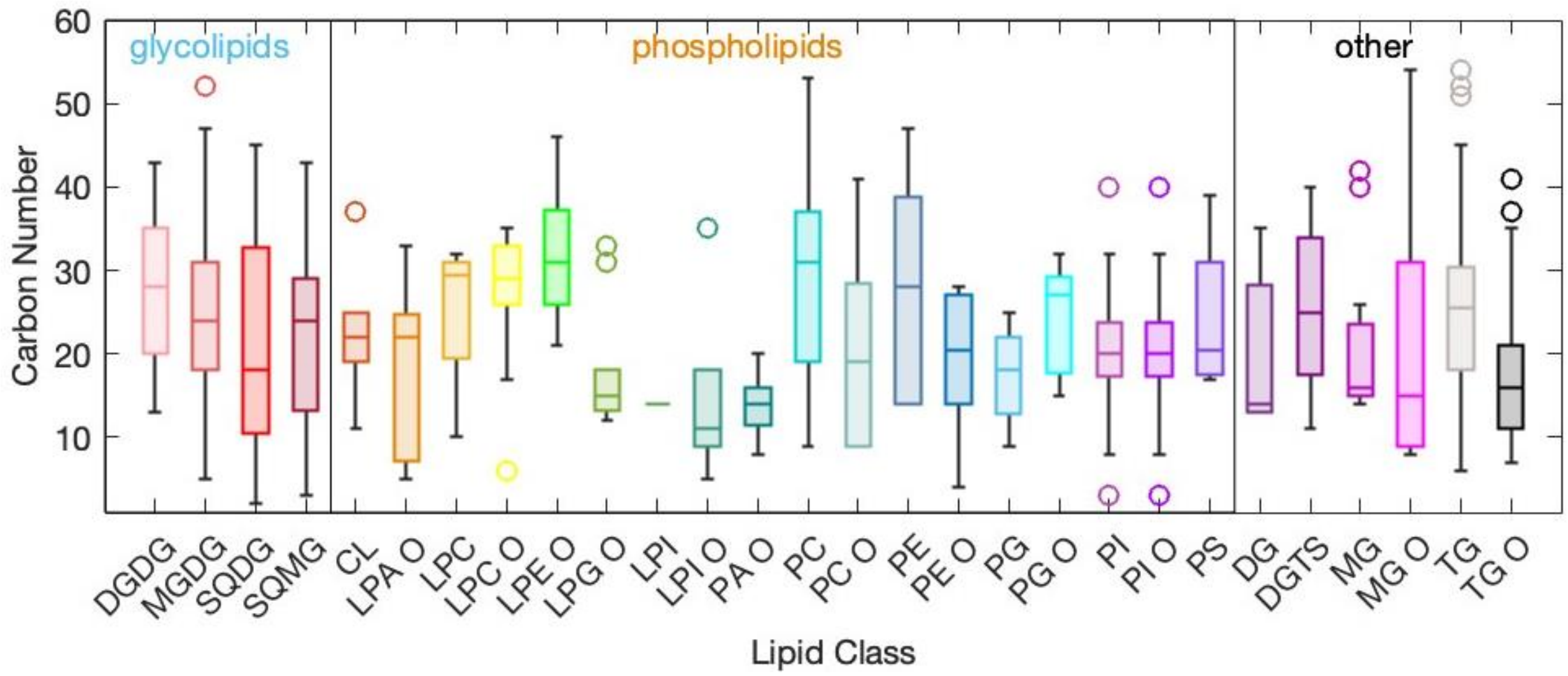

Figure S3. The number of carbons in the combined fatty acid chains per lipid, grouped by the lipid classes identified. Bars are ordered the same as those in Figure 3.

References

(1) Agblemanyo, F. E.; Frossard, A. A.; Wozniak, A. S.; D., A.; Birt, A. E. The Rosette-Based Glass Plate (RGP) Sampler: A Cost-Effective Technique for High-Volume, Low-Thickness Sea Surface Microlayer Collection. *Limnol. Oceanogr.:Methods*. 2026, e70081. DOI: 10.1002/lom3.70081.

(2) *PubChem*. National Library of Medicine, https://pubchem.ncbi.nlm.nih.gov/ (accessed 2026).

(3) Van Mooy, B. A. S.; Fredricks, H. F. Bacterial and Eukaryotic Intact Polar Lipids in the Eastern Subtropical South Pacific: Water-column Distribution, Planktonic Sources, and Fatty Acid Composition. *Geochim. Cosmochim. Acta* **2010**, *74* (22), 6499-6516. DOI: https://doi.org/10.1016/j.gca.2010.08.026.

(4) Miranda, M. L.; Mustaffa, N. I. H.; Robinson, T. B.; Stolle, C.; Ribas-Ribas, M.; Wurl, O.; Zielinski, O. Influence of Solar Radiation on Biogeochemical Parameters and Fluorescent Dissolved Organic Microlayer of the Southern Coastal North Sea. *Elem. Sci. Anth.* **2018**, *6*. DOI: 10.1525/elementa.278.

(5) Cunliffe, M.; Upstill-Goddard, R. C.; Murrell, J. C. Microbiology of Aquatic Surface Microlayers. *FEMS Microbiol. Rev.* **2011**, *35* (2), 233-246. DOI: 10.1111/j.1574-6976.2010.00246.x.

(6) Bligh, E. G.; Dyer, W. J. A Rapid Method of Total Lipid Extraction and Purification. *Can. J. Biochem. Physiol.* **1959**, *37* (8), 911-917. DOI: 10.1139/o59-099.
